# Reducing Dietary Acid With Fruit and Vegetables Versus Oral Alkali in People With Chronic Kidney Disease (ReDACKD): A Clinical Research Protocol

**DOI:** 10.1177/20543581231190180

**Published:** 2023-08-07

**Authors:** Rebecca Mollard, Katrina Cachero, Bohdan Luhovyy, Heather Martin, Sharon Moisiuk, Sepideh Mahboobi, Robert Balshaw, David Collister, Leah Cahill, Karthik K. Tennankore, Navdeep Tangri, Dylan MacKay

**Affiliations:** 1 Department of Food and Human Nutritional Sciences, Faculty of Agriculture and Food Science, University of Manitoba, Winnipeg, Canada; 2 Chronic Disease Innovation Centre, Seven Oaks General Hospital, University of Manitoba, Winnipeg, Canada; 3 Department of Applied Human Nutrition, Mount Saint Vincent University, Halifax, NS, Canada; 4 Department of Internal Medicine, University of Manitoba, Winnipeg, Canada; 5 Department of Community Health Sciences, University of Manitoba, Winnipeg, Canada; 6 Division of Nephrology, Department of Medicine, University of Alberta, Edmonton, Canada; 7 Department of Medicine, Dalhousie University, Halifax, NS, Canada; 8 Nova Scotia Health Authority, Dalhousie University, Halifax, NS, Canada

**Keywords:** chronic kidney disease, metabolic acidosis, oral alkali therapy, sodium bicarbonate

## Abstract

**Background:**

Individuals with chronic kidney disease (CKD) can develop metabolic acidosis which, in turn, is associated with faster progression of CKD and an increased need for dialysis. Oral sodium bicarbonate (the current standard of care therapy for metabolic acidosis) is poorly tolerated leading to low adherence. Base-producing or alkalizing Fruit and vegetables have potential as an alternative treatment for metabolic acidosis as they have been shown to reduce acid load arising from the diet.

**Objective:**

This trial will evaluate the feasibility of providing base-producing fruit and vegetables as a dietary treatment for metabolic acidosis, compared with oral sodium bicarbonate.

**Design:**

A 2-arm, open-label, dual-center, randomized controlled feasibility trial.

**Setting:**

Two Canadian sites: a nephrology clinic in Winnipeg, Manitoba, and a nephrology clinic in Halifax, Nova Scotia.

**Participants:**

Adult participants with G3-G5 CKD and metabolic acidosis.

**Measurements:**

Participants will undergo baseline measurements and attend 5 study visits over 12 months at which they will have a measurement of feasibility criteria as well as blood pressure, blood and urine biochemistry, 5-repetition chair stand test (STS5), and questionnaires to assess quality of life and symptoms. Furthermore, participants fill out Automated Self-Administered 24-hour recalls (ASA-24) in the beginning, middle, and end of trial.

**Methods:**

A total of 40 eligible participants will be randomized 1:1 to either base-producing fruit and vegetables (experimental) group or sodium bicarbonate (control) group, beginning from a daily dose of 1500 mg.

**Limitations:**

Using self-administered dietary assessments, lack of supervision over the consumption of study treatments and the possible disappointment of the control group for not receiving fruit and vegetables would be considered as limitations for this study. However, we are planning to undertake proper practices to overcome the possible limitations. These practices are discussed throughout the article in detail.

**Conclusions:**

This study will generate data on base-producing fruit and vegetables consumption as a dietary treatment for metabolic acidosis in CKD. The data will be used to design a future multi-center trial looking at slowing CKD progression in people with metabolic acidosis.

**Trial Registration:**

This study is registered on clinicaltrials.gov with the identifier NCT05113641.

## Abrégé

**Contexte:**

Les personnes atteintes d’insuffisance rénale chronique (IRC) courent le risque de développer une acidose métabolique, laquelle est associée à une progression plus rapide de l’IRC et à un besoin accru de dialyse. La prise de bicarbonate de sodium par voie orale (la norme actuelle de traitement de l’acidose métabolique) est mal tolérée, ce qui se traduit par une faible adhérence. Les fruit et légumes basiques ou alcalifiants ont un potentiel de traitement alternatif pour l’acidose métabolique, car il a été démontré qu’ils peuvent réduire la charge acide provenant de l’alimentation.

**Objectif:**

cet essai permettra d’évaluer la faisabilité d’un traitement alimentaire de l’acidose métabolique, en misant sur la consommation de fruit et légumes basiques ou alcalifiants, par rapport à la prise de bicarbonate de sodium par voie orale.

**Type d’étude:**

essai de faisabilité contrôlé, randomisé, ouvert, à deux bras, mené dans deux centres.

**Cadre:**

deux sites canadiens, soit une clinique de néphrologie à Winnipeg (Manitoba) et une autre à Halifax (Nouvelle-Écosse).

**Sujets:**

des patients adultes atteints d’IRC de stade G3-G5 et d’acidose métabolique.

**Mesures:**

les participants seront soumis à des mesures initiales et devront se présenter à cinq visites d’étude réparties sur 12 mois. Au cours de chacune, les patients subiront une mesure des critères de faisabilité, une mesure de la pression artérielle, un bilan sanguin et urinaire, un test de lever de chaise à cinq répétitions (STS5 — *Five Times Sit to Stand Test*) et devront répondre à des questionnaires évaluant la qualité de vie et les symptômes. Les participants devront également utiliser un outil en ligne de rappels alimentaires de 24 heures autoadministrés et automatisés (ASA24 — Automated Self-Administered 24-hours) au début, à mi-parcours et à la fin de l’essai.

**Méthodologie:**

40 patients admissibles seront randomisés (1:1) dans le groupe expérimental (fruit et légumes basiques ou alcalifiants) ou dans le groupe témoin (bicarbonate de sodium) avec une dose quotidienne initiale de 1 500 mg.

**Limites:**

l’utilisation d’outils d’évaluation alimentaire autoadministrés, le manque de supervision de la consommation des traitements à l’étude et la possible déception du groupe témoin de ne pas recevoir de fruit et légumes constituent des limites pour cette étude. Nous prévoyons cependant adopter des pratiques appropriées pour surmonter ces possibles limites. Ces pratiques sont discutées plus en détail dans le manuscrit.

**Conclusion:**

cette étude produira des données sur la consommation de fruit et légumes basiques ou alcalifiants comme traitement alimentaire pour l’acidose métabolique en contexte d’IRC. Ces données seront utilisées pour concevoir un futur essai multicentrique visant à ralentir la progression de l’IRC chez les personnes atteintes d’acidose métabolique.

**Enregistrement de l’essai:**

Cette étude a reçu l’approbation du Conseil d’éthique de la recherche en santé de l’Université du Manitoba (HS24768 [B2021:025]) et est enregistrée sur ClinicalTrials.gov avec l’identifiant NCT05113641.

## Introduction

### Background and Rationale

Chronic kidney disease (CKD) is a major public health problem with a rising incidence and prevalence worldwide. Metabolic acidosis is a common complication of advanced CKD (category G3b-G4) resulting from a decline in the kidney’s ability to excrete excess dietary acid and is typically diagnosed by a serum bicarbonate level of less than 22 mEq/L. International practice guidelines recommend that individuals with CKD and metabolic acidosis be treated with oral alkali therapy such as sodium citrate or sodium bicarbonate (NaHCO_3_), when appropriate, to help maintain a serum bicarbonate level >22 mEq/L.^1^ However, treatment rates have been reported to be as low as 10%^2^ as the treatment can be poorly tolerated leading to its discontinuation.^3^ If sodium bicarbonate is used as oral alkali therapy, the doses required to treat acidosis entail up to 12 pills a day with substantial sodium load, which can lead to worsening albuminuria and potentially negate the efficacy of renin-angiotensin-aldosterone system (RAAS) inhibitors,^4^ which are important disease-modifying treatments for CKD.

Base-producing fruit and vegetables (F+V) could be a potential treatment for metabolic acidosis as they reduce the dietary contribution to overall acid load that must be managed by the kidneys.^5-8^

Kidneys regulate acid-base homeostasis and healthy kidneys excrete approximately 60 mmol/L daily dietary acid load derived from a typical Western diet.^9^ The most common way to measure the dietary impact on acid/base balance is the potential renal acid load (PRAL).^5^ Using the Remer and Manz equation, foods with negative PRAL values are base-producing and contribute to dietary bicarbonate equivalents. F+V have been shown to have negative PRAL values as their consumption produces more base precursors than acid precursors.^5,10^ Previous studies have shown beneficial effects of F+V supplementation on metabolic acidosis and markers of kidney injury^10,11^; F+V supplementation could also preserve renal function better than oral alkali, possibly due to better control of blood pressure.^10^ These findings, however, were from single-center studies conducted in a specific patient population in Texas, USA, and have not been evaluated in a multicenter randomized controlled trial in Canada.

### Objectives

The objective of this study is to evaluate the feasibility of base-producing F+V consumption for metabolic acidosis treatment in people with CKD across 2 nephrology centers in Canada, while also assessing physical function and nutrition outcome measures. The data generated by this feasibility study will be used to inform a future definitive multicenter trial looking at the efficacy of F+V treatment in CKD progression in people with metabolic acidosis.

### Trial Design

ReDACKD is a dual-center, 2-arm, 1 to 1 parallel group, randomized controlled feasibility trial. The primary outcome of feasibility will be assessed at trial completion (12 months). Following baseline assessments, secondary outcomes will be assessed quarterly, at months 3, 6, 9, and 12.

## Participants, Interventions, and Outcomes

### Study Setting

This protocol has been reported in compliance with the Standard Protocol Items: Recommendations for Interventional Trials (SPIRIT) guideline.^12^ This study will take place at the nephrology clinic at Seven Oaks General Hospital (SOGH) in Winnipeg, Manitoba, Canada, and the nephrology clinic in the Dickson building at the QEII Health Sciences Centre in Halifax, Nova Scotia, Canada.

Details regarding inclusion and exclusion criteria are listed in Table 1. Participants have the right to withdraw from the study at any time. Furthermore, the investigator may decide to discontinue a participant from the trial at any time due to specific criteria. Withdrawal criteria are elaborated in Table 1.

**Table 1.** Eligibility and Withdrawal Criteria.

Eligibility criteria		Withdrawal criteria
Inclusion criteria	Exclusion criteria	
*The study will include participants who:* ○ are able to communicate in English and willing/able to provide informed consent to participate in the trial; ○ are aged 18 years or above ○ have an eGFR between 15 and 40 mL/min/1.73 m^2^ ○ have 2 consecutive measurements of serum bicarbonate of 14 to 22 mEq/L within 6 months, with or without sodium bicarbonate therapy ○ have systolic and diastolic blood pressure <160/100 mm Hg, serum potassium <5.3 mmol/L, and hemoglobin A1C (HbA1C) below ≤11% ○ are registered in the multidisciplinary nephrology clinics in Winnipeg or Halifax	Participants will be excluded if they: ○ have a history of anuria, dialysis, or acute kidney injury/acute kidney failure in the 3 months prior to screening ○ have a diagnosis of chronic obstructive pulmonary disease that requires the participant to be on oxygen ○ have NYHA Class 3-4 Heart Failure symptoms ○ are a heart, liver, or kidney transplant recipient ○ had a myocardial infarction or stroke within the last 6 months ○ are unable to consume study treatments or control (such as swallowing or gastrointestinal issues) ○ have participated in another research trial involving an investigational product in the past 12 weeks ○ are currently on potassium binding therapy ○ are pregnant or lactating	○ As per participants’ decision^a^ ○ As per investigator’s decision for the following reasons: ● Pregnancy ● Ineligibility (either arising during the trial or retrospectively having been overlooked at screening) ● Persistent hyperkalemia thought to be due to the intervention and not amenable to potassium-binding therapy ● Disease progression which results in inability to continue to comply with the protocol ● Loss to follow-up

*Note.* eGFR = estimated glomerular filtration rate; NYHA = New York Heart Association.

a Participants have the right to withdraw from the study at any time; these participants are requested to contact a research team member to inform them their decision.

Discontinuation of an intervention will not result in exclusion of the data for that participant from analysis as the primary analysis will be based on an intention-to-treat, but there will also be a completer only analysis performed. If a participant is withdrawn within the first 4 weeks of the trial, they will be replaced. If the replacement participant is also withdrawn, there will be no subsequent replacement. If provided, the reason for withdrawal will be recorded in the case report form (CRF).

The research team includes 2 partners who have lived experience in health care and personal experience managing CKD. The partners were involved in preparation of the grant application, the trial protocol, trial education materials, and one partner is a member on the steering committee and advises on the conduct of the feasibility trial in various capacities. The decision to undertake this research was also informed by people with lived experience of CKD who identified nutrition interventions as important to this disease in focus groups which were conducted as part of a Preparing for Research by Engaging Patient and Public Partners (PREPPP) Award funded and conducted in collaboration with the George and Fay Yee Center for Healthcare Innovation, the Manitoba CIHR SPOR SUPPORT unit.^13^

### Consenting Process

#### Who will take informed consent?

The participant must personally sign and date the latest approved version of the Informed Consent form via paper copy or through the REDCap online platform, hosted at the University of Manitoba, prior to the baseline assessment. Participants will be asked to attend a virtual or in-person consent visit. Coordinators or graduate students who have completed Good Clinical Practice (GCP) training and have been trained on the procedure will obtain informed consent.^14^

No additional consent is being obtained because no ancillary studies or studies involving biological specimens are planned.

## Interventions

### Intervention Description

#### Fruit and vegetables (experimental group)

Participants will receive weekly deliveries of fresh, frozen, and/or dried F+V, as well as juices and soups, all selected for their negative PRAL values^5^ and shelf-life. Each delivery will contain enough F+V so that the participant and all members of their household can have the daily recommended intake (30-40 mEq per day), a list can be found in Supplemental Appendix A. In Winnipeg, the food deliveries will be completed by the Save-On-Foods grocery store, who have contributed to the costs of the deliveries to this research program and who are providing the F+V at cost. In Halifax, the F+V will be sourced from various food wholesalers and retailers and the deliveries will be conducted by the staff working at the Department of Applied Human Nutrition at Mount Saint Vincent University. In Halifax, Noggins Corner Farm will be donating the apples for the study.

Participants will receive a 1-hour dietary counseling in the first week from a registered dietitian (RD), via in-person or videoconference depending on participant preference. In this session, the RD will recommend the best ways to prepare and include the F+V into the participant’s current diet. Delivery contents will be adjusted based on the participants’ preferences and medical condition. High glycemic choices such as fruit juices will not be provided to those with diabetes mellitus (DM). Participants will also be provided with a handbook (Supplemental Appendix B) including all information they need. All participants in the experimental group will be started at a F+V intake equal to 30 to 40 mEq per day in reduction in dietary acid load estimated by the PRAL equation (Supplemental Appendix A). Bioavailability of bicarbonate related to whole food containing a variety of nutrients, fiber, and other substances is considered. This dose is based on the milliequivalent dose of oral alkali therapy that would be achieved with 3000 mg of sodium bicarbonate a day, well within the normal range used to treat metabolic acidosis in CKD.^15^ If the participants’ 1- and 3-month serum bicarbonate concentrations are <22 mEq/L, we will have their recommended amount of F+V increased to 40 to 50 mEq per day, and if these values exceed 29 mEq/L, their target dose of F+V in milliequivalents per day will be reduced by 25%. Dietary adaptation will be the first response to metabolic acidosis; however, if adjustments in bicarbonate from diet are unable to fix the acidosis, then the study physician will determine whether the participant can continue in the study. To minimize the potential risk of hyperkalemia, defined as serum potassium ≥5.5 mEq/L in the intervention arm, F+V boxes will be restricted to contain less than 400 mg of potassium per 100 g serving. In participants who develop hyperkalemia, the F+V content will be further restricted to foods which are even lower in potassium (<200 mg per 100 g). Any hyperkalemia, refractory to dietary management, will be treated with potassium binders per standard of care at each site.

#### Sodium bicarbonate (control group)

Our comparator is oral sodium bicarbonate therapy, the current standard treatment for the management of metabolic acidosis in people with CKD in Canada. Participants randomized to this group will receive oral sodium bicarbonate 500 mg tablets 3 times a day, reflecting a common starting dose at clinical practice.^16^ At each site, a study nephrologist will prescribe the oral alkali therapy. The medication will be dispensed by SOGH pharmacy in Winnipeg and a registered nurse study coordinator will dispense the medications in Halifax. Following the principles of a pragmatic standard care control, decisions around dose titration for the sodium bicarbonate will then be transferred to the participant’s nephrologist who will be responsible for monitoring the participant’s serum bicarbonate concentration to maintain a serum bicarbonate level >22 mEq/L. In addition, all participants will receive standard dietary counseling that they would normally receive during their interdisciplinary CKD clinic from an RD as part of their standard care, but the counseling about incorporating low PRAL foods will only be provided to F+V group during the study RD counseling session.

#### Adherence to the interventions

Compliance to F+V intake will be evaluated through 24-hour dietary recalls. There will be a study dispensing log for weekly box deliveries, and additionally, research personnel will keep in touch with participants over the phone, on a weekly basis, throughout the study, using a short script (Supplementary File 1).

Compliance in control group will be evaluated by pill counts and a sodium bicarbonate pill distribution log will be completed for participants in this group. At each study visit, participants will be reminded about the importance of adhering to their intervention.

### Relevant Concomitant Care Permitted or Prohibited During the Trial

Participants who are already undergoing potassium-binding therapy will not be recruited into this trial. Participants in the trial will continue to receive CKD care by their nephrologist during the trial, except for oral sodium bicarbonate therapy if enrolled in the F+V group.

### Provisions for Post-Trial Care

Participants will continue to be followed by their existing nephrology care teams post trial.

### Outcomes

The primary outcomes of this study are intended to help inform the design of a future trial and hypothesized effect to better inform elements such as recruitment (patient interest), retention strategies (motivation), and effectiveness of fruits and vegetables in management of metabolic acidosis.^17^ Thus, feasibility will be demonstrated if all of the following occur:

- Ratio of randomized to eligible participants is greater than 50%, calculated among all eligible patients approached to participate.
- Average recruitment rate of at least 0.5 participants per center per week of active recruitment based on typical presentation of eligible patients seen in nephrology clinic and an estimation that 25% of them will be interested to participate in trial. Actual recruitment rate will be evaluated at end of the trial.
- Follow-up with at least 95% of participant outcomes to evaluate patient amenability to comply and effectiveness of intervention is expected based on previous experiences with trials similar to this.
- Average adherence to F+V intervention over 75% of participants, adherence will be defined as the participant achieving at least a 25% reduction in dietary PRAL per day, according to the average PRAL over the three 24-hour recalls (at 6- and 12-month assessments) versus baseline assessment.

The secondary outcomes of this study will be as follows.

#### Quality of life

Assessing physical functioning includes the 5-repetition chair stand test (STS5), which measures the fastest time taken to stand 5 times from a chair with arms folded.^18^ This test provides health care professionals with a reliable measure of physical functioning in people with CKD and has been used in previous studies as part of frailty outcomes and participants’ physical functioning in CKD.^19-22^ In addition, participants will be required to fill out the health-related quality of life using the physical function domain of the Kidney Disease Quality of Life Short Form (KDQOL-SF) questionnaire (Supplemental Appendix C), which assesses typical daily activities, for example, running, lifting heavy objects, moving a table, using a vacuum cleaner, or climbing stairs. A second questionnaire, the Edmonton symptom assessment revised renal (ESAS:r) (Supplemental Appendix D), will also be filled out by participants. This questionnaire is used to assess how participants are feeling at the time of assessment and includes symptoms commonly encountered by those with advanced CKD, namely, nausea, fatigue, restless legs, lack of appetite, and so on. These will be assessed at 3, 6, 9, and 12 months.

#### Clinical chemistry

Blood and urine samples will be collected and the following will be measured in blood samples: albumin, blood urea nitrogen (BUN), bicarbonate, calcium, chloride, creatinine, estimated glomerular filtration rate (eGFR), glucose, phosphorus, potassium, sodium, and Hemoglobin A1C (HbA1C). For the urine sample, urine albumin-to-creatinine ratio (uACR) will be measured at baseline and at 3, 6, 9, and 12 months.

#### Blood pressure

An oscillometric blood pressure monitor will be used to collect the systolic and diastolic blood pressure and heart rate. At rest, the measures will be taken in triplicate on the non-dominant arm and assessed at baseline and at 3, 6, 9, and 12 months.

#### Hospitalization and mortality

Information on recent hospitalizations and mortality from participant’s clinical records will be collected.

#### Nutrition

To assess nutrition intake, participants will be required to fill out 3-day Automated Self-Administered (ASA) 24-hour diet recalls online (at least 2 weekdays and 1 weekend day), via the ASA-24 Web site.^23^ Participants will be given instructions on how to fill these out and will be given a paper copy to fill out if unable to complete online (Supplemental Appendix E). These recalls will be used to assess dietary PRAL and will be collected at baseline and at 6 and 12 months.

#### Height and weight

Anthropometric measurements, such as height and weight, will be measured using a weighing scale stadiometer. This will be assessed at baseline and 12 months.

### Participant Timeline

Trial flowchart is demonstrated in Supplemental Appendix F.

### Sample Size

This study will recruit 40 participants, 20 at each site. The sample size for each treatment and for each site is within the 20 to 27 range of sample sizes recommended by current best practice for feasibility studies.^24^

### Recruitment

Research coordinators will work with an individual within the circle of care (such as a nurse, clinical clerk, or physician) of patients attending the Manitoba or Halifax renal programs interdisciplinary CKD clinics to pre-identify potentially eligible participants. At both sites, there are approximately 40 clinic visits per week for patients with CKD stages G3-G5. Based on a chart review, it is estimated that 10 patients per week will have evidence of metabolic acidosis, 4 will meet the eligibility criteria, and at least 2 will be randomized per week at each center. Therefore, we anticipate reaching target enrollment within 12 weeks once both sites have started.

### Assignment of Interventions

#### Randomization

To improve balance and to minimize the possibility of bias, the randomization sequence will be generated by a third-party biostatistician at the George and Fay Yee Center for Health Innovation. Randomization will be performed using code written in the R statistical programming language (Version 3.5.3). Treatments will be randomized by site, assigned with a 1:1 ratio of F+V to sodium bicarbonate treatment groups, with random block sizes of 2 and 4 at each site.

The randomization sequence, will be put into a secure web-based REDCap tool, hosted by the University of Manitoba, by the data management staff, and will be not visible to study coordinators or other study staff.

Allocation to either study groups will be performed by a trial coordinator through REDCap tool using the sequence created in R and otherwise will be concealed from the study team. The study coordinators will be the ones enrolling participants.

#### Blinding

Blinding of participants and study staff is not possible given the nature of the intervention. Assessors who complete in-person assessments will be blinded to the intervention assignment. Participants will be asked not to communicate their intervention to the assessor. Furthermore, the study statistician and the entire data management team will be blinded to allocation until the database has been locked and reviewed for quality prior to the final analysis.

### Data Collection and Management

#### Plans for assessment and collection of outcomes

Data will be entered into REDCap database. The Data Coordinating Centre within the Data Science group at the Centre for Healthcare Innovation will undertake the data management in REDCap database and conduct regular data quality checks.

#### Plans to promote participant retention and complete follow-up

Participants will be receiving ongoing CKD care, and changes in care locations in later stage CKD are uncommon. Based on previous trials in similar populations at these sites, we expect a loss to follow-up of <5% at 1 year.^25,26^

#### Data management

A unique numerical coding system will be implemented that will not contain any direct identifiers, such as names or initials to de-identify the data collected in this study. All research data collected throughout the study will be entered into the study database using this unique code. All research records will be kept for 10 years, in keeping with site protocols. After 10 years, paper files will be disposed of using the confidential document destruction method. Electronic data will be de-identified and retained for 10 years following the study completion.

### Confidentiality

The trial staff will ensure that the participants’ anonymity is maintained. The participants will be identified only by a participant ID number on all trial documents (except the consent form and the study master list) and any electronic database. All documents will be stored securely and only accessible by trial staff and authorized personnel. The trial will comply with The Personal Health Information Act (PHIA) or The Freedom of Information and Protection of Privacy Act (FIPPA) of Manitoba and the Nova Scotia Freedom of Information and Protection of Privacy Act (FOIPOP). Electronic data may also be shared, as indicated in the protocol and consent form, in de-identified form to academic journals for publication purposes. This data may be stored by the academic journal or other open-access repositories under an open-access policy in which case it may be used by other researchers for further data analysis and research purposes.

### Plans for Collection, Laboratory Evaluation, and Storage of Biological Specimens for Genetic or Molecular Analysis in This Trial/Future Use

Study samples will not be stored for any longer than 10 years following the end of the research project, nor shared with any other group, other than what is indicated in the protocol and the consent form. After 10 years, all samples will be destroyed.

### Statistical Methods

#### Statistical methods for primary and secondary outcomes

For this study, the feasibility outcomes will be summarized overall and by group and compared to the pre-specified thresholds in the outcomes section above. The influence of sex on feasibility measures will be investigated using *t*-tests or chi-square test. Secondary outcomes and other measures will be summarized overall, by group, by study center and by sex using conventional descriptive statistics (means, medians, quantiles, and standard deviations, as well as counts and percentages). The feasibility data will be examined by the study leadership for evidence of issues with the study design or logistics and how these issues may affect the larger definitive confirmatory trial. For the secondary outcomes, such as STS5, KDQOL-SF, serum bicarbonate, eGFR, and uACR, the effects of treatment will be examined using a linear mixed-effects model for repeated measures. Sex will be included in this model as a fixed factor. Model assumptions for outcome data will be verified by visual inspection of residual plots and through sensitivity analyses regarding missing value strategies. Outcomes that appear to violate the modeling assumptions may be transformed prior to analysis or analyzed using another, more appropriate model (eg, generalized linear mixed models) in supportive analyses. Demographic data will be reported as the mean ± standard deviation. Analyses of the secondary outcomes will be reported as unadjusted and adjusted (expected marginal) means with 95% confidence intervals. In general, analyses resulting in *P*-values less than .05 will be described as statistically significant, without adjustment for multiple inference. All results arising from secondary, exploratory, and supportive analyses will be identified as such.

There are no plans for interim analyses.

#### Methods for additional analyses

The primary analysis will be conducted using the *All Participants* analysis set using treatments as assigned (ie, following intention to treat). The primary analysis will be repeated in the *Completers* analysis set. Demographics and all other baseline measurements will be summarized by treatment and by site in the All Participants set as well as in the Completers set.

*Completers* Analysis Set: All participants who have completed the trial and provided required outcome data on pre-specified study follow-up visits will be considered as completers. All Participants Analysis set will be all participants initially randomized to study arms.

#### Methods in analysis to handle protocol non-adherence and any statistical methods to handle missing data

As a component of the primary feasibility analyses, the number and proportion of missing values will be documented in the clinical study report. In general, missing values will not be imputed unless otherwise noted. Analyses will exclude data from participants who have missing values for any variable required for the analysis. When data are observed to be unusual in a way that cannot be explained or ruled to be in error, analyses may be repeated after excluding the record involved. These additional analyses will be presented as sensitivity analyses. All protocol deviations documented in the clinical trial database will be tabulated (if appropriate) and listed in the clinical study report. Any changes made to this statistical analysis plan will be documented in the same way as protocol amendments. Any variations from this plan will be documented as such in the clinical study report. Missing value imputation methods such as multiple imputation may be used in sensitivity analyses if the presence of missing values may have substantive effect on the conclusions.

### Plans to Give Access to the Full Protocol, Participant Level Data, and Statistical Code

After the study is concluded, there are plans to share the anonymized data set (upon submission of request to the principal investigator, agreement of the trial management, and completion of a data transfer agreement). Statistical analysis code will also be made available upon request and uploaded in Supplementary Material in any results manuscripts.

### Oversight and Monitoring

#### Composition of the coordinating care center and trial steering committee

The trial will be coordinated by the Chronic Disease Innovation Center (CDIC) at Seven Oaks Hospital (https://changinghealthcaredelivery.ca/). Research coordinators will be responsible for daily coordination of the project at each site, including project set-up, recruitment, scheduling the participants and deliveries, and data collection. Grocers and/or research staff will perform the assembly and delivery of the fruit and vegetables. Study dietitians at CDIC (Winnipeg) and Dalhousie/Appetite Lab (Halifax) will be responsible for nutritional education and recommendations in the fruit and vegetables arm. Drs Tangri and Tennankore will provide the oral alkali prescriptions and act as the qualified investigators in Winnipeg and Halifax, respectively. The trial steering committee will include Drs Mackay, Tangri, Tennankore, Cahill, Balshaw, Luhovyy, Mollard, and Moisiuk. This steering committee will meet quarterly to discuss and monitor the trial progress. The steering committee will also discuss and approve the protocol and any amendments. The steering committee will also review reports and recommendations from the data safety monitoring board (DSMB).

#### Composition of the data monitoring committee, its role, and reporting structure

DSMB will include 2 nephrologists from outside the study team, as well as an independent statistician. The DSMB will conduct a safety review of the study when half the participants have finished 6 months of the intervention, and then every 6 months until study completion. The DSMB will focus on participant safety, but will also review study progress and conduct. As this is a feasibility trial, there will be no pre-specified stopping criteria.

### Adverse Event Reporting and Harms

Given the nature of the interventions, it is unlikely that any adverse events (AEs) will be related to the trial. However, all AEs occurring during the trial that are observed by the investigators or reported by the participants will be recorded on the CRF, whether or not attributed to trial intervention. The following information will be recorded: description, date of onset and end date, severity, and assessment of relatedness to trial intervention. Follow-up information should be provided as necessary. In case any AE is reported, participants will be offered to be seen in the next available clinic visit or within 1 week, whichever earlier, and will continue to be followed in the clinic until the AE is resolved. The severity of events will be assessed on the following scale: 1 = mild, 2 = moderate, and 3 = severe. Adverse events considered related to the trial intervention as judged by a Qualified Investigator will be followed either until resolution or the event is considered stable. In case AEs result in withdrawal from the trial, the patients who are withdrawn due to adverse treatment reaction will also be followed by the CKD clinic until the AEs has resolved.

### Frequency and Plans for Auditing Trial Conduct

Regular monitoring will be performed according to GCP by the principal investigators or a delegate. A subset of all data will be evaluated for compliance with the protocol and accuracy in relation to source documents. Following written standard operating procedures, the monitoring will verify that the clinical trial is conducted and data are generated, documented, and reported in compliance with the protocol, GCP, and the applicable regulatory requirements.

### Research Ethics Approval

ReDACKD has received approval from the University of Manitoba Health Research Ethics Board HS24768 (B2021:025), the Nova Scotia Health Research Ethics Board (#1027715), and the Mount Saint Vincent University Research Ethics Board (#2021-194). All amendments to this study’s protocol are reviewed and approved by the University of Manitoba Bannatyne Research Ethics Board, the Nova Scotia Health Research Ethics Board, and the Mount Saint Vincent University Research Ethics Board, and changes to the protocol are updated on Clinicaltrials.gov.

## Discussion

It is estimated that 1 in 5 people with eGFR of 30 to 44 mL/min/1.73 m^2^ have metabolic acidosis, and this increases to 1 in 3 as eGFR decreases to 15 mL/min/1.72 m^2^.^27^ This represents approximately 350 000 Canadians.^28-30^ In several large cohort studies, metabolic acidosis is associated with a higher risk of CKD progression and mortality,^31,32^ increased cardiovascular disease (CVD) risk, and the higher burden of frailty.^33,34^ In people receiving oral alkali therapy in Manitoba, median time to discontinuation was 6 months, and more than two thirds of people (68%) had discontinued therapy at 1 year.^35^ In a recent large randomized controlled trial, oral sodium bicarbonate was ineffective for treating metabolic acidosis or slowing CKD progression.^3^ This finding was potentially due to the high dropout rate in the trial, with 20% withdrawal due to patient choice in sodium bicarbonate arm.

This dual-center feasibility trial will be the first randomized trial in Canada to evaluate the feasibility of F+V provision as a treatment for CKD and metabolic acidosis. This trial is critical in designing the next step of our research program with the goal of a pan-Canadian phase 3 trial testing the efficacy of base-producing F+V provision on slowing the progression of CKD. Similar studies have been conducted previously in Texas, USA. In 2013, Goraya et al showed that 12 months provision of F+V improved metabolic acidosis and reduced kidney injury in individuals with stage 4 CKD.^11^ The same group randomized participants with stage 3 CKD into 3 groups, usual care, sodium bicarbonate, and F+V for 36 months^36^ and showed that both sodium bicarbonate therapy and base-producing F+V could preserve eGFR in this individuals. The authors also suggested considering F+V as an alternative to sodium-based alkali therapy for metabolic acidosis in individuals with low GFR. These researchers followed the participants for 60 months^10^ and concluded that improvement in metabolic acidosis and preserving eGFR were comparable in F+V and sodium bicarbonate group, but F+V arm showed better improvement in CVD risk indicators.

These trials carefully selected participants without DM, CVD, edema, or hyperkalemia. As such, more than 90% of Canadian people with CKD and metabolic acidosis would have been excluded from these studies,^35^ rendering them non-generalizable and highlighting the need for feasibility testing in a clinically relevant patient population in Canada prior to a large-scale definitive trial to evaluate the intervention on CKD progression. The longest time for F+V delivery in these studies was 60 months. We will be leveraging existing grocery store home delivery infrastructure in our trial; therefore, we are confident that our trial will be able to feasibly deliver the F+V intervention for the planned 12 months and that infrastructure exists or could be built to make this intervention realistic in practice.

We propose that adding F+V that have negative PRAL values would be more effective in improving acid-base balance than modifying dietary protein type or intake. This is based on our belief that a single clear intervention, like adding F+V, would be more easily implemented than a more complex intervention such as modifying protein sources, and that adding something is likely easier than restricting. Furthermore, in a previous study conducted by our research group,^13^ we found that many people with late-stage CKD already do not achieve the recommended intakes for protein and fiber. Hence, reducing the intake of high-quality protein might not be appropriate, whereas adding F+V could improve the overall dietary quality and nutrient intakes.

This study is not without limitations; an automated self-administered 24-hour dietary assessment tool will be used for measurement of dietary intake, which is reliant on respondent’s memory.^17^ The consumption of F+V and sodium bicarbonate tablets is not supervised; however, we are planning to constantly check in with participants and make appropriate adjustments. The control group might be disappointed for not receiving fruits and vegetables; however, all participants will be remunerated for getting involved in the study.

## Supplemental Material

sj-docx-1-cjk-10.1177_20543581231190180 – Supplemental material for Reducing Dietary Acid With Fruit and Vegetables Versus Oral Alkali in People With Chronic Kidney Disease (ReDACKD): A Clinical Research Protocol Click here for additional data file. Supplemental material, sj-docx-1-cjk-10.1177_20543581231190180 for Reducing Dietary Acid With Fruit and Vegetables Versus Oral Alkali in People With Chronic Kidney Disease (ReDACKD): A Clinical Research Protocol by Rebecca Mollard, Katrina Cachero, Bohdan Luhovyy, Heather Martin, Sharon Moisiuk, Sepideh Mahboobi, Robert Balshaw, David Collister, Leah Cahill, Karthik K. Tennankore, Navdeep Tangri and Dylan MacKay in Canadian Journal of Kidney Health and Disease

sj-docx-2-cjk-10.1177_20543581231190180 – Supplemental material for Reducing Dietary Acid With Fruit and Vegetables Versus Oral Alkali in People With Chronic Kidney Disease (ReDACKD): A Clinical Research Protocol Click here for additional data file. Supplemental material, sj-docx-2-cjk-10.1177_20543581231190180 for Reducing Dietary Acid With Fruit and Vegetables Versus Oral Alkali in People With Chronic Kidney Disease (ReDACKD): A Clinical Research Protocol by Rebecca Mollard, Katrina Cachero, Bohdan Luhovyy, Heather Martin, Sharon Moisiuk, Sepideh Mahboobi, Robert Balshaw, David Collister, Leah Cahill, Karthik K. Tennankore, Navdeep Tangri and Dylan MacKay in Canadian Journal of Kidney Health and Disease
