# Supplementary material for: Reducing Dietary Acid With Fruit and Vegetables Versus Oral Alkali in People With Chronic Kidney Disease (ReDACKD): A Clinical Research Protocol
Source: Can J Kidney Health Dis. 2023 Aug 7;10:20543581231190180. doi: 10.1177/20543581231190180 (PMC10408321; doi:10.1177/20543581231190180)
Supplement: sj-docx-1-cjk-10.1177_20543581231190180 – Supplemental material for Reducing Dietary Acid With Fruit and Vegetables Versus Oral Alkali in People With Chronic Kidney Disease (ReDACKD): A Clinical Research Protocol [file sj-docx-1-cjk-10.1177_20543581231190180.docx]

**Appendices**

**Appendix A: Food list of low acid fruits and vegetables**

*The participant will eat a 30-40-point dose of low acid fruit and vegetables per day. Each food is measured in grams.*

| **Low acid Foods** | | **Dose sizes in grams** | | |
| --- | --- | --- | --- | --- |
|  |  | **20**  **point dose** | **10**  **point dose** | **5**  **point dose** |
| **Vegetables** | Beets | 158 | 79 | 40 |
|  | Buttercup squash | 328 | 164 | 82 |
|  | Butternut squash | 257 | 128 | 64 |
|  | Carrots | 226 | 113 | 56 |
|  | Celery | 393 | 196 | 98 |
|  | Turnip, cooked | 418 | 209 | 104 |
|  | Eggplant | 622 | 311 | 155 |
|  | Green peppers | 579 | 289 | 145 |
|  | Hubbard squash | 239 | 120 | 60 |
|  | Jicama | 592 | 296 | 148 |
|  | White flowered squash | 440 | 220 | 110 |
|  | Pumpkin | 435 | 218 | 109 |
|  | Turnip, raw | 395 | 197 | 99 |
|  | Red peppers | 496 | 248 | 124 |
|  | Red radish | 391 | 196 | 97 |
|  | Spaghetti squash | 686 | 343 | 172 |
|  | Yam | 388 | 194 | 97 |
|  | Yellow peppers | 476 | 238 | 119 |
|  | Zucchini | 323 | 161 | 81 |
| **Fruits** | Apples | 467 | 233 | 117 |
|  | Blackberries | 482 | 241 | 120 |
|  | Cantaloupe | 247 | 123 | 62 |
|  | Banana, fresh | 244 | 122 | 61 |
|  | Mango, fresh | 402 | 201 | 101 |
|  | Tomato, fresh | 270 | 135 | 67 |
|  | Berry mix, frozen | 745 | 373 | 172 |
|  | Grapefruit | 261 | 130 | 65 |
|  | Kiwi, green | 516 | 258 | 129 |
|  | Honeydew | 235 | 117 | 59 |
|  | Nectarines | 293 | 146 | 73 |
|  | Oranges | 300 | 150 | 75 |
|  | Peaches | 287 | 143 | 72 |
|  | Pears | 411 | 206 | 103 |
|  | Raspberries | 675 | 338 | 169 |
|  | Seedless grapes | 410 | 205 | 103 |
|  | Strawberries | 517 | 259 | 129 |
| **Soups** | Gazpacho soup | 220 | 110 | 55 |
|  | Tomato soup | 208 | 104 | 52 |
|  | Vegetable soup | 260 | 130 | 65 |
| **Milk and Juices** | Almond milk | 229 | 115 | 57 |
|  | Carrot juice | 167 | 83 | 42 |
|  | Cranberry juice | 209 | 105 | 52 |
|  | Grapefruit juice | 264 | 132 | 66 |
|  | Vegetable juice | 110 | 55 | 28 |
|  | Apple juice | 404 | 202 | 101 |
|  | Prune juice | 157 | 79 | 39 |
|  | Tomato juice | 207 | 104 | 52 |

**Appendix B. Participant Handbook**


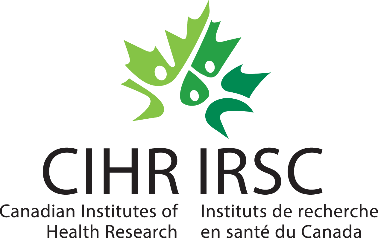

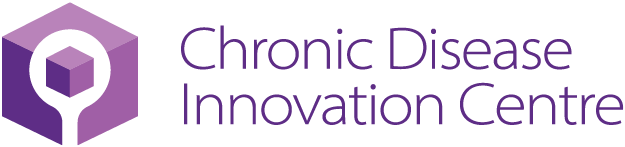

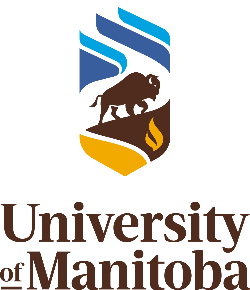


ReDACKD Study

# Fruit and Vegetable Study Group

# Start date: ____________

# Exit date: _____________

Welcome

You are in the fruit and vegetable group.

You will receive weekly boxes of fruits and vegetables. The boxes will be delivered to your home and will contain a mix of fresh, frozen, and dried fruits and vegetables, as well as juices and soups. The foods in the box will vary each week based on what foods are available. In this booklet, you will find everything you need to be in the study. If you have any questions and/or concerns, please contact the study coordinator at
[REDACKDTRIAL@umanitoba.ca](mailto:REDACKDTRIAL@umanitoba.ca).

In this booklet, you will find the following:

1. A checklist for fruit and vegetable box deliveries
2. Appointment with Dietitian – Notes page
3. Study information – What is potential renal acid load? How much fruits and vegetables do I eat per day?
4. A list of low acid fruits and vegetables
5. How to measure food when using a bowl or plate
6. Examples of how much fruits and vegetables to eat per day
7. Example recipes
8. Different ways to cook vegetables
9. Frequently Asked Questions

Fruit and Vegetable Box Deliveries CHECKLIST

You will be receiving boxes every week for the next 12 months. Please check off boxes as you receive them.

| 1 | 2 | 3 | 4 | 5 | 6 | 7 | 8 | 9 | 10 |
| --- | --- | --- | --- | --- | --- | --- | --- | --- | --- |

| 11 | 12 | 13 | 14 | 15 | 16 | 17 | 18 | 19 | 20 |
| --- | --- | --- | --- | --- | --- | --- | --- | --- | --- |

| 21 | 22 | 23 | 24 | 25 | 26 | 27 | 28 | 29 | 30 |
| --- | --- | --- | --- | --- | --- | --- | --- | --- | --- |

| 31 | 32 | 33 | 34 | 35 | 36 | 37 | 38 | 39 | 40 |
| --- | --- | --- | --- | --- | --- | --- | --- | --- | --- |

| 41 | 42 | 43 | 44 | 45 | 46 | 47 | 48 | 49 | 50 |
| --- | --- | --- | --- | --- | --- | --- | --- | --- | --- |

| 51 | 52 |
| --- | --- |

Appointment with Dietitian

For the first week of the study you will be meeting with a Dietitian, they will help recommend the best ways to prepare and include the fruit and vegetables in your diet while also following any recommendations given to you by your nephrologist. Please use this space to take notes for yourself when meeting with the dietitian.

Date: _____________________________

Notes: _____________________________________________________________________ ____________________________________________________________________________

____________________________________________________________________________

____________________________________________________________________________

____________________________________________________________________________

____________________________________________________________________________

____________________________________________________________________________

____________________________________________________________________________

____________________________________________________________________________

____________________________________________________________________________

____________________________________________________________________________

____________________________________________________________________________

____________________________________________________________________________

____________________________________________________________________________

# What is dietary potential renal acid load (PRAL)?

To help us measure how food affects your body’s acid/base balance, we are using a method called potential renal acid load (also known as PRAL). PRAL is the amount of acid your body produces when you eat certain foods. When we eat higher PRAL foods, our body produces more acid. We want to reduce the amount of acid our body produces because when our kidneys don’t work well, they have troubles keeping the right balance of acid in our body. In this study, you will be eating a daily dose of low acid (low PRAL) foods. We are looking at how much fruits and vegetables it takes to lower your acid load.

# How much fruits and vegetables do I eat per day?

We have created a point system that represents the dosages for the fruits and vegetables. You will eat a 30–40-point dose of low acid fruits and vegetables throughout the day. We have included a table on **page 7** that shows a list of fruits and vegetables grouped by different dosages.

This picture below shows what 30 points throughout the day looks like.


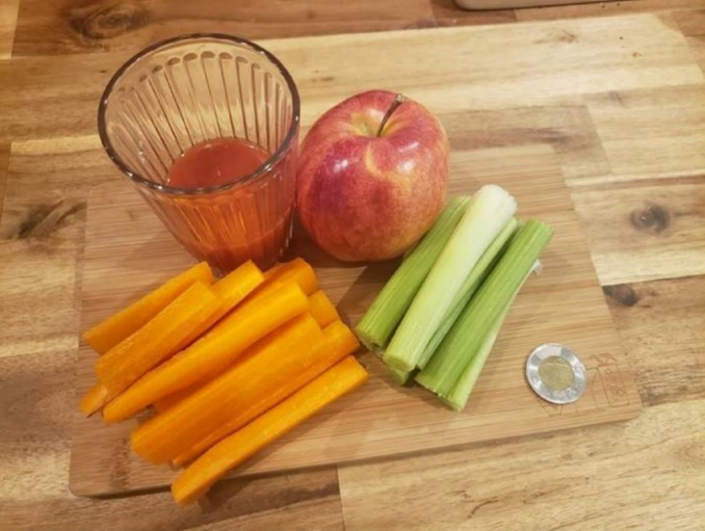


This includes 1 medium apple, a half cup of tomato juice, 100 grams of carrots, and 100 grams of celery.

There will be more examples shown on **page 12**.

**** $2 coin in photo is to give an idea of food portion size**

| Food list of low acid fruits and vegetables This is a list of low acid fruits and vegetables. You will eat a **30-40 point dose** of low acid fruits and vegetables per day. Each food will be measured in grams. Examples of different combinations are on page 12. | | | | |
| --- | --- | --- | --- | --- |
| **Low acid Foods** | | **Dose sizes in grams** | | |
|  |  | **20**  **point dose** | **10**  **point dose** | **5**  **point dose** |
| **Vegetables** | Beets | 158 | 79 | 40 |
|  | Buttercup squash | 328 | 164 | 82 |
|  | Butternut squash | 257 | 128 | 64 |
|  | Carrots | 226 | 113 | 56 |
|  | Celery | 393 | 196 | 98 |
|  | Turnip, cooked | 418 | 209 | 104 |
|  | Eggplant | 622 | 311 | 155 |
|  | Green peppers | 579 | 289 | 145 |
|  | Hubbard squash | 239 | 120 | 60 |
|  | Jicama | 592 | 296 | 148 |
|  | White flowered squash | 440 | 220 | 110 |
|  | Pumpkin | 435 | 218 | 109 |
|  | Turnip, raw | 395 | 197 | 99 |
|  | Red peppers | 496 | 248 | 124 |
|  | Red radish | 391 | 196 | 97 |
|  | Spaghetti squash | 686 | 343 | 172 |
|  | Yam | 388 | 194 | 97 |
|  | Yellow peppers | 476 | 238 | 119 |
|  | Zucchini | 323 | 161 | 81 |
| **Fruits** | Apples | 467 | 233 | 117 |
|  | Blackberries | 482 | 241 | 120 |
|  | Cantaloupe | 247 | 123 | 62 |
|  | Banana, fresh | 244 | 122 | 61 |
|  | Mango, fresh | 402 | 201 | 101 |
|  | Tomato, fresh | 270 | 135 | 67 |
|  | Berry mix, frozen | 745 | 373 | 172 |
|  | Grapefruit | 261 | 130 | 65 |
|  | Kiwi, green | 516 | 258 | 129 |
|  | Honeydew | 235 | 117 | 59 |
|  | Nectarines | 293 | 146 | 73 |
|  | Oranges | 300 | 150 | 75 |
|  | Peaches | 287 | 143 | 72 |
|  | Pears | 411 | 206 | 103 |
|  | Raspberries | 675 | 338 | 169 |
|  | Seedless grapes | 410 | 205 | 103 |
|  | Strawberries | 517 | 259 | 129 |
| **Soups** | Gazpacho soup | 220 | 110 | 55 |
|  | Tomato soup | 208 | 104 | 52 |
|  | Vegetable soup | 260 | 130 | 65 |
| **Milk and Juices** | Almond milk | 229 | 115 | 57 |
|  | Carrot juice | 167 | 83 | 42 |
|  | Cranberry juice | 209 | 105 | 52 |
|  | Grapefruit juice | 264 | 132 | 66 |
|  | Vegetable juice | 110 | 55 | 28 |
|  | Apple juice | 404 | 202 | 101 |
|  | Prune juice | 157 | 79 | 39 |
|  | Tomato juice | 207 | 104 | 52 |

Measuring FoodS

# How to use the scale

It is important to keep the scale clean and free of any food before you use it as this can affect the weight.

***Measuring foods with a bowl or plate***

| 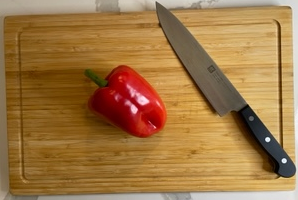 | **STEP 1.**  Get a cutting board, a small to medium sized plate, knife, and your fruit or vegetable of choice. |
| --- | --- |
| 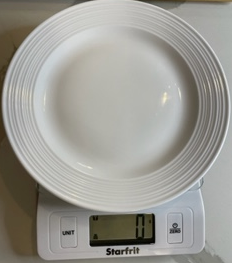  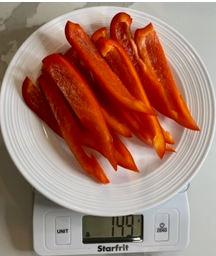 | **STEP 2.**  Turn the scale on by pressing ‘ON’.  Place the plate on the scale. After you have placed the plate on the scale, the number on the scale will change. This number is how much your plate weighs.  ***This is the most important step***  **To make sure you are only measuring the food’s weight (and not also the weight of the plate), press ‘TAR’ before adding any food onto the plate.**  **Once the scale says 0, you are ready to add your food of choice to the plate.**  On your cutting board, cut the fruit or vegetable of choice to a certain size, like in strips shown in the picture.  Place the fruit or vegetable on the plate to see how much it weighs.  Cut OR add pieces of the fruit or vegetable until you get the desired weight. |
| 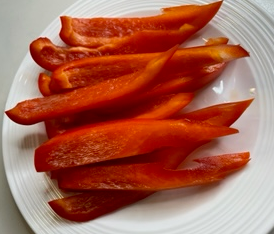 | **STEP 3.**  Remove the plate from the scale and now you are ready to eat! |
| **STEP 4.**  To measure another food product, follow steps 1-4. | |

# Examples of food combinations to eat a 30-40 point dose

There are different ways to get 30 to 40 points in a day. You can eat 2 types of foods throughout the day or even up to 6 different types of foods. It is all up to you. These are just some example combinations. If you have any questions, please contact the study coordinator.

**** $1 coin in photos is to give an idea of food portion size ****

# Getting 30-40 points from 2 foods in a day

208 grams of tomato soup (20 points) with a 122 gram banana (10 points)

- Instead of the banana, you can also have 115 grams of almond milk

# Getting 30-40 points from 3 foods in a day

260 grams of vegetable soup (20 points) with a 117 gram apple (5 points) and 57 grams of almond milk (5 points)

- Instead of the apple and almond milk, you can also have 122 gram banana or 123 grams of cantaloupe

# Getting 30-40 points from 4 foods in a day

115 grams of almond milk (10 points) with 209 grams of cooked mashed turnip (10 points), 129 grams of strawberries (5 points) and a 72 gram peach (5 points)


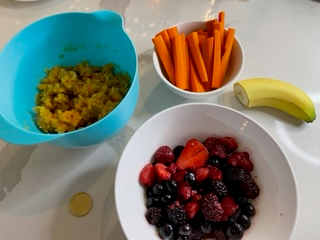


1. 194 grams of cooked mashed yam (10 points)

2. 113 grams of carrots (10 points)

3. 186 grams of frozen berry mix (5 points)

4. 61 gram banana (5 points)

# Getting 30-40 points from 5 foods in a day

** Keep in mind that you don’t have to eat all these at once, you can eat them throughout the day.

1. 343 grams of spaghetti squash (10 points) or 40 grams of beets (5 points)

2. 67 grams of fresh tomatoes (5 points)

3. 72 grams of peaches (5 points)

4. 103 gram pear (5 points)

5. 103 grams of seedless grapes (5 points)

Different ways to cook vegetables

# What are some low PRAL foods you can roast/bake?

*Examples: squash, beets, radish, yams, bell peppers, and zucchini.*

1. Slice your veggie(s) of choice evenly. If you are using a mix of different vegetables, typically root vegetables like squash, yam, carrots take longer to cook than softer vegetables like eggplant or zucchini.
2. Preheat oven to 400^o^F or 425^o^F. Put the slices of veggies on a baking sheet or pan (doesn’t matter what size – as long as you are able to spread the veggies evenly across it). Avoid filling the pan too high! This can cause them to cook unevenly.
3. Season with salt and pepper or your seasoning of choice (can use dried or fresh herbs). Drizzle with olive oil (make sure to coat all of the veggies).
4. Put in oven at 400^o^F to 425^o^F and toss the veggies to make sure they cook evenly (cooking time depends on type of veggie being roasted and the size – typically we want the veggies tender enough to pierce with a fork).

# What are some food items you can steam?

*Examples: zucchini, carrots, celery, potatoes and radishes.*

To steam vegetables, you need a steamer basket or a steamer pan.

1. Slice your veggie evenly. The smaller the pieces, the shorter the cooking time.
2. Fill the pot with water under the steamer basket then bring it to a boil.
3. Once boiling, add the veggies to a steamer basket and cover the pot with a lid. Cooking times can vary from 5 to 12 minutes, to check if it’s cooked test a piece with a fork). Once it is cooked, carefully remove the veggies from the basket and eat them plain or toss them with some olive oil and salt or your favourite spices.

# What are some food items you can grill?

*Examples: squash, beets, radish, yams, bell peppers, zucchini, apples, pears, nectarines, mango and peaches.*

To grill fruits or vegetables, you will need a barbecue grill. A grilling basket may make it easier for grilling.

1.
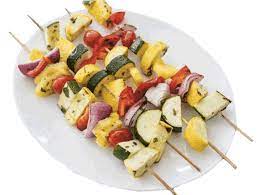
Cut your veggie or fruit to your liking (typically for fruits, you want them cut in large chunks where for vegetables, can be cut however you’d prefer). There is also the option of adding them into a skewer (picture shown on the right).
2. Once you have cut up the fruit or vegetable, they need to be brushed with a neutral oil (example: vegetable oil, canola oil) that can withstand high heat.
3. Grill the fruit or vegetable over high heat (fruits will be ready in less than 5 minutes where vegetables will take longer than 5 minutes).

example Recipes

These recipes are examples only.
You don’t have to make or eat these specific ones.

# Fruit Salad

**Prep time:** 15 minutes **Makes 8 to 10 servings**

*For this recipe, you need:* a large bowl, cutting board, knife

| Ingredients:  For the dressing –   - ¼ cup of honey - ¼ cup orange juice - Zest of 1 lemon | For the salad –   - 1lb strawberries - 6 oz raspberries - 1 orange, peeled, wedges cut in half - 2 apples, peeled, chopped into squares - 2 cup grapes, sliced in half - 1 mango, peeled and chopped |
| --- | --- |

Instructions:

1. In a small bowl, whisk together honey, orange juice, and lemon zest. Add the fruit to a large bowl and pour over dressing, tossing gently to combine. Store in fridge covered until ready to serve.

*Note: This recipe is good for up to 5 days in the fridge but tastes best when enjoyed within 3 days.

# Making a smoothie

**Prep time:** 10 minutes **Makes 1 to 2 servings**

*For this recipe, you need:* a blender

Step 1: Choose a liquid (almond milk or water). Start with 1 cup. How much liquid you use will determine your smoothies’ texture and thickness.

*Add more liquid for a thin smoothie; add less if you want it thicker, like a frozen yogurt. Adding ice can create a milkshake-like texture.

Step 2: Add 2 cups of fruit. Fruit can add sweetness and texture. Both fresh and frozen fruit will work but frozen fruit gives a more refreshing zing and thicker consistency.

*Different combinations can include:*

1. 2 cups frozen berry mix 2. 1 banana, ½ mango, 1 peach

Step 3: You can also add a few tablespoons of peanut butter, yogurt, ice cubes, chia seeds, or oats. *OPTIONAL*

Step 4: If you find that the smoothie doesn’t taste sweet enough to your liking, you can add other things like honey, sugar/stevia, vanilla/almond extract, or cinnamon. 1-2 teaspoons should do the trick!

Step 5: Blend it all up until you get the consistency you want.

Enjoy!

# Mashed parsnips and carrots

**Prep time:** 10 minutes **Cook time:** 20 minutes **Makes 3 cups**

*For this recipe you need:* cutting board, knife, large pot, large bowl, steamer basket (optional), strainer, potato masher

Ingredients:

- 4 large carrots, peeled & cut into 1-inch long chunks (about 3 cups)
- 4 large parsnips, peeled & cut into 1-inch long chunks (about 3 cups)
- 2 tablespoons extra virgin olive oil
- ½ teaspoon salt
- ¼ teaspoon pepper
- Pinch nutmeg
- Freshly chopped dill for garnish (optional)

Instructions:

1. If you have a steamer basket, choose a pot that fits the basket. If you don’t, that’s ok. Bring 5 cups of water to a boil in a large pot. Make sure that the pot isn’t overcrowded, if it is, switch to a larger pot.
2. Add carrots and parsnips to the pot or the steamer basket.
3. Let it cook until the veggies are tender, usually about 18-20 minutes – can be tested with a fork, it should slide in & come out easily.
   1. If steaming – remove the basket from the pan and put veggies in a large bowl.
   2. If boiling – place the vegetables in a strainer over the sink to get rid of the water. Use the strainer to add the vegetables into a large bowl.
4. Add in the extra virgin olive oil, salt, pepper, and a pinch of nutmeg to the veggies. Mash the veggies with a potato masher.
5. Once the veggies reach a creamy-like texture. You can serve it hot with a sprinkle of fresh dill on top. Enjoy!

F.A.Q.

# Do I have to cook the food?

It is recommended that you cook (if needed). There are a variety of foods on the list that don’t require any cooking.

# Can I eat more than 30 to 40 points of vegetables and fruits per day?

As long as you have eaten 30-40 points for the day, you are more than welcome to eat more, there are no restrictions.

# What do I do if I am eating at a restaurant or at someone else’s’ house?

If you are eating at a restaurant or at someone else’s’ house, eat the fruits or vegetables prior to or after the event so you aren’t worried or thinking about it during.

# What do I do if I go on holidays or travel outside the city?

If you travel outside of the city, please let the study coordinator know. This can be accommodated with proper preparation and communication with the coordinator.

# What do I do if I am advised by my doctor to limit my fluids?

If you are on a fluid restriction, please review this with the study dietitian or coordinator to ensure you are not going over your fluid limit.

# What do I do if I have other nutrition recommendations given to me by my nephrologist or dietitian?

Please follow any nutrition recommendations given to you by your nephrologist or dietitian, if you have any concerns, please let the study coordinator know.

# 23.If you have any other study-related questions, please contact the study coordinator at [REDACKDTRIAL@umanitoba.ca](mailto:REDACKDTRIAL@umanitoba.ca)

**Appendix C: Health related Quality of Life (QOL) using the physical function domain of the Kidney Disease Quality of Life Short Form (KDQOL-SF) questionnaire**

**Appendix D: Edmonton Symptom Assessment System Revised Renal Questionnaire adapted from Alberta Health Services (ESAS:r)**


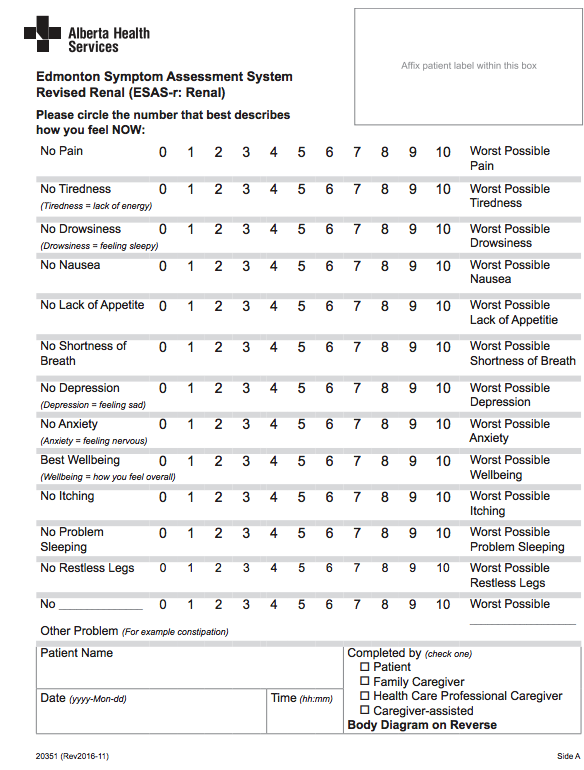


**Appendix E: Automated Self-Administered 24-hour Canada (ASA24®) dietary assessment tool**

| 24-hour food Log  **Participant ID:** | | | | | |
| --- | --- | --- | --- | --- | --- |
| **Day ---:** | | | | | |
| Time | Location (home, restaurant, work, etc.) | Were you watching TV or on your phone? | Were you eating alone or with someone else? Please state who (partner, co-worker, etc.) | Food (please include all information such as method of cooking, ingredients, etc.) | Amount (in cups, tbsp., tsp., pcs, etc.) |
|  |  |  |  |  |  |
| Time | Location (home, restaurant, work, etc.) | Were you watching TV or on your phone? | Were you eating alone or with someone else? Please state who (partner, co-worker, etc.) | Food (please include all information such as method of cooking, ingredients, etc.) | Amount (in cups, tbsp., tsp., pcs, etc.) |
|  |  |  |  |  |  |
| Time | Location (home, restaurant, work, etc.) | Were you watching TV or on your phone? | Were you eating alone or with someone else? Please state who (partner, co-worker, etc.) | Food (please include all information such as method of cooking, ingredients, etc.) | Amount (in cups, tbsp., tsp., pcs, etc.) |
|  |  |  |  |  |  |

**Appendix F: Trial Flow Chart**

## Enrollment

**Eligibility assessment**

**Excluded:**

- Individuals do not meet the inclusion /exclusion criteria
- Declined to participate

**Randomization sequence and Allocation (n: 40)**

20 participants in Halifax and 20 participants in Winnipeg

CY

**Fruit and Vegetables (F+V)**

(n=20)

- Weekly home delivery of F+V box
- Dietitian support
- Standard ongoing multidisciplinary CKD care

**Sodium bicarbonate therapy**

(n=20)

- Standard ongoing multidisciplinary CKD care
- Oral sodium bicarbonate therapy

## Data Analysis

**Safety assessment at 1 month:**

- Clinical chemistry
- Blood pressure

**Outcomes assessed at baseline, 3, 6, 9 and 12 months**

- Chair stand time
- Health related quality of life
- Edmonton symptom assessment system revised renal
- Clinical chemistry
- Blood pressure
- Dietary intake (baseline, 6 and 12 months)

One year
