# Supplementary material for: Reducing Dietary Acid With Fruit and Vegetables Versus Oral Alkali in People With Chronic Kidney Disease (ReDACKD): A Clinical Research Protocol
Source: Can J Kidney Health Dis. 2023 Aug 7;10:20543581231190180. doi: 10.1177/20543581231190180 (PMC10408321; doi:10.1177/20543581231190180)
Supplement: sj-docx-2-cjk-10.1177_20543581231190180 – Supplemental material for Reducing Dietary Acid With Fruit and Vegetables Versus Oral Alkali in People With Chronic Kidney Disease (ReDACKD): A Clinical Research Protocol [file sj-docx-2-cjk-10.1177_20543581231190180.docx]

*Weekly follow up of food deliveries (F+V arm)*

*Date: Participant ID: Staff name:*

- Did you receive the fruit and vegetable delivery?
- Were all the foods acceptable in terms of quality?
- Did you like the items in your box? Is there any item that you want us to remove from or add to your box for the next time?
- Where you able to consume the recommended daily amounts?
- Do you have any questions or additional comments?
